# When sequential use of mepolizumab and dupilumab in a severe atopic eosinophilic asthmatic questions the role of eosinophils in mediating the clinical expression of the disease: a case report

**DOI:** 10.1186/s13256-023-04255-8

**Published:** 2024-01-31

**Authors:** M. Sabbe, F. Schleich, P. Janssens, R. Louis

**Affiliations:** 1 Department of Respiratory Medicine, CHU Liege, Liège, Belgium; 2 Dermatology, Medicard, Libramont, Belgium

**Keywords:** Asthma, Mepolizumab, Dupilumab, Eosinophils, FeNO, IgE

## Abstract

**Background:**

The advent of biologics has resulted in major progress in the treatment of severe T2 high asthmatics. There are currently several classes of biologics approved for severe asthma including anti-immunoglobulin E, anti-interleukin-5/interleukin 5R, anti-interleukin 4/interleukin 13R, and anti-thymic stromal lymphopoietin.

**Case presentations:**

Here we report the case of a 55-year-old Caucasian man with severe eosinophilic atopic asthma, who sequentially benefited from a treatment with mepolizumab, an anti-interleukin-5 monoclonal antibody, followed by treatment with dupilumab, an anti-interleukin-4/interleukin-13R antibody, the switch being justified by a flare-up of dermatitis while on mepolizumab. Overall, the patient has been followed for 72 months, including 42 months on mepolizumab and 30 months on dupilumab. Close monitoring of exacerbations, asthma control, lung function, asthma quality of life, and biomarkers shows that both biologics reduced asthma exacerbation and provided an improvement in asthma control and quality of life, with the patient achieving remission after 30 months on dupilumab. However, the effects of the two biologics on the biomarkers were very different, with mepolizumab controlling eosinophilic inflammation and dupilumab reducing serum immunoglobulin E and fractional exhaled nitric oxide levels.

**Conclusion:**

The originality of this case resides in the description of clinical status and biomarker evolution after a sequential use of mepolizumab and dupilumab in a severe atopic eosinophilic asthmatic. It shows that mepolizumab reduces exacerbation and improves asthma control by curbing eosinophilic inflammation whereas dupilumab provides asthma remission without controlling airway eosinophilic inflammation.

## Introduction

Severe asthma features a chronic airway inflammation mediated by many interleukins [1, 2]. The advent of biologics directed toward T2 cytokines has changed the course of the disease and the quality of life of many severe asthmatics qualified as being T2 high patients. There are currently five classes of biologics approved for treating severe asthma, including anti-immunoglobulin E (anti-IgE) (omalizumab), anti-interleukin (IL)-5 (mepolizumab, reslizumab), anti-IL5R (benralizumab), anti-IL4/IL13R (dupilumab), and anti-thymic stromal lymphopoietin (TSLP) (tezepelumab) [3–5]. They all have different mechanisms of action and some of them have treatment indications other than for sole severe asthma such as chronic idiopathic urticaria for anti-IgE [6], atopic dermatitis for dupilumab [7], and nasal polyposis for omalizumab, mepolizumab, and dupilumab [8, 9]. Mepolizumab is a humanized monoclonal antibody (IgG1, kappa) directed against interleukin-5, while dupilumab is a recombinant human monoclonal antibody (IgG4) that blocks the alpha chain of the receptor of interleukin-4 and interleukin-13. Mepolizumab decreases blood and lung eosinophils and reduces asthma exacerbations [10] and the use of systemic corticoids as maintenance treatment, whereas the main action of dupilumab, which also reduces exacerbation and oral corticosteroid (OCS) burden, seems to be related to airway smooth muscle relaxation [11] and reduction in mucus hypersecretion [12].

Here we report the case of a severe eosinophilic atopic asthmatic who sequentially benefited from a treatment with mepolizumab and dupilumab, in whom the switch was justified by a flare-up of dermatitis. This case illustrates the different mechanisms of action between the two biologics and raises questions about the role of airway eosinophils in mediating clinical expression of asthma.

## Case report

The Caucasian patient living in Belgium was born in 1951 and was referred to our asthma clinic at the age of 65 years.

His asthma started at the age of 55 years after a clinical history of a nasal polyposis for more than 10 years. He had smoked 10 cigarettes/day until the age of 34 years and had carried out the job of hairdresser until the age of 60 years. An available biology at asthma onset when the patients was 55 years old revealed high total serum IgE at 522 kU/l and eosinophilia at 540/µl, representing 7.8% of blood leucocytes.

Our patient had been followed in primary care setting for 10 years for his asthma, had been treated by high-dose inhaled corticoids (ICS) combined to long acting β2-agonists (LABA) (dipropionate fluticasone/salmeterol 500/50 µg 2 × 1/24 hour) and montelukast 10 mg/day. While controlled for 7 years with this treatment, the patient started to show signs of clinical deterioration later in the course of the disease. Indeed, despite the high-intensity treatment, the patient received multiple courses of OCS to relieve symptoms of breathlessness and cough over the past 3 years prior to the visit at our asthma clinic. More precisely, the patient reported nine courses of methylprednisolone in the 12 months prior to the visit at our asthma clinic, but no lung function had been performed.

The first visit at our asthma clinic gave the results described in Table 1. The patient was atopic with multiple sensitizations toward common aeroallergens and had a high total serum IgE at 1510 kU/l. He displayed increased fractional exhaled nitric oxide (FeNO) at 86 ppb, increased sputum eosinophil count at 8%, but normal blood eosinophil count at 239 µl. He showed airflow limitation (pre-bronchodilation FEV1 67% predicted and FEV1/FVC 76% and post-bronchodilation FEV1 73% predicted. As a reminder FEV1 measures the forced expiratory volume in one second and FVC measures the forced vital capacity,) and severe bronchial hyperresponsiveness to methacholine with a provocative concentration causing a 20% fall in FEV1 measured at 0.17 mg/ml. Asthma was uncontrolled with an asthma control test (ACT) at 8 and an asthma control questionnaire (ACQ) at 4.29. Asthma quality of life was poor with an Asthma Quality of Life Questionnaire (AQLQ) at 3.07.

**Table 1:** Patient at his first assessment at our asthma clinic

Demographic characteristics	
Age (years) Gender BMI Profession	63 Male 28 kg/m^2^ Hairdresser
Airway and blood inflammatory markers	
FeNO Sputum eosinophil count Blood eosinophil count Total IgE	63 ppb 8% 239/μl 1510 kU/l
Patient-reported outcomes Quality of life Control of his asthma	
ACT ACQ AQLQ	8 4.29 3.07
Respiratory function assessment	
VEMS pre-bronchodilation VEMS post-bronchodilation VEMS/CVF % of reversibility with salbutamol PC 20 Methacholine	67% of predicted values 73% of predicted values 76% 6% 0.17 mg/ml
Comorbidity	
Nasal polyposis	
Atopic status	
Dpt Grass Cat Dog Birch	2.6 kU/l 3.4 kU/l < 0.35 kU/l 1 kU/l 2.8 kU/l

*BMI* Body Mass index; *FeNO* fraction of exhaled nitric oxide; *ACT* asthma control test; *ACQ* asthma control questionnaire; *AQLQ* asthma quality of life questionnaire

The patient was then included in a trial investigating the value of triple therapy (ICS, Long-acting beta-agonists (LABA) and Long-acting muscarinic antagonists (LAMA)) in patients uncontrolled by high doses ICS/LABA (IRIDIUM study, a randomised, double-blind, controlled phase 3 study [13]) but was soon discarded because of poor asthma control and two exacerbations requiring courses of OCS within the first 2 months of the trial. Two blood eosinophil counts sampled 1 month apart during the exacerbation phases revealed values of 620 µl and 600 µl, respectively. During the second exacerbation, FEV1 was measured at 41% predicted, rising to 57% predicted 15 min after 400 µg inhaled salbutamol.

Therefore, on the basis of recurrent exacerbations (nine over the last 12 months) imposing courses of OCS and the lack of improved control while being scrutinized in a randomized controlled trial (RCT) assessing the value of triple therapy in a severe eosinophilic patient, we decided to start mepolizumab 100 mg subcutaneous once per month, which had just been made available and reimbursed by public health authorities in Belgium by this time. The last course of OCS was terminated the week before mepolizumab initiation. The day of the initiation of mepolizumab, we measured FEV1 at 64% predicted, FEV1/FVC ratio at 63%, and FEV1 rose up to 92% predicted after inhalation of 400 µg salbutamol. FeNO was measured at 45 ppb, sputum eosinophil count at 46%, and blood eosinophil at 443 µl.

**Fig. 1:**
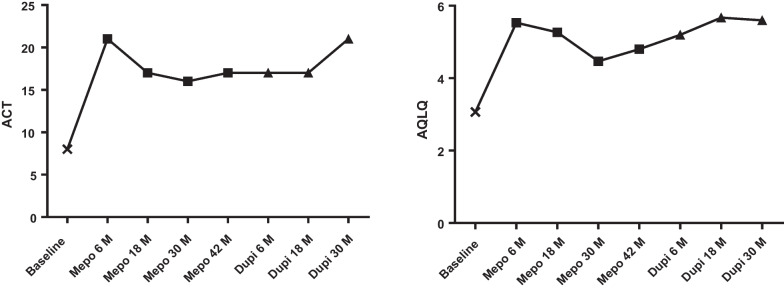
Evolution of asthma control and quality of life, solid squares indicate mepomizumab period and solid triangles indicate dupilumab period

**Fig. 2:**
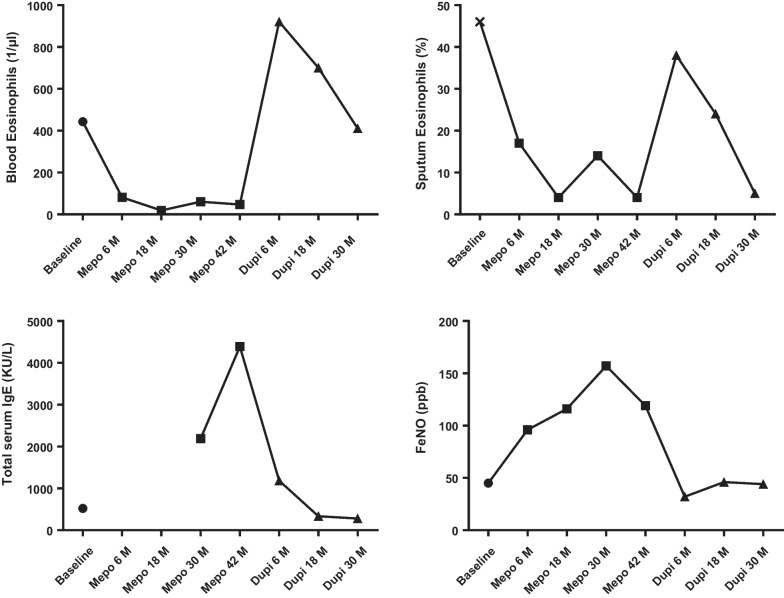
Evolution of the inflammatory markers, solid square indicates mepomizumab period and solid triangle indicates dupilumab period

After initiation of mepolizumab we observed an improvement in asthma control and asthma quality of life (Table 2, Fig. 1) together with a clear reduction in exacerbation rate over the next 42 months, with no asthma exacerbation at all for the time period between 18 months and 42 months (Table 2). Blood and sputum eosinophils markedly decreased and remained low across the whole time period when patient received mepolizumab. On the contrary, FeNO and total serum IgE levels increased (Table 2, Fig. 2). Patient stopped treatment with montelukast but kept receiving fluticasone/salmeterol 1000/100 µg/day. Asthma was not fully controlled yet, with ACT and ACQ values of 17 and 2.43, respectively.

**Table 2:** Timeline of the control, quality of life and perspective of the patient, with the inflammatory and the lung function values

	ACT	ACQ	AQLQ	FeNO (ppb)	IgE ( kU/l)	Blood eosinophils (mm^3^)	Sputum eosinophils (%)	Exacerbations previous 12 months	FEV_1_ Pre-bronch (%)	FEV_1_ Post-bronch (%)
First visit at asthma clinic	8	4.29	3.07	86	1510	239	8	9	67	73
Two weeks after exacerbation treated by OCS	17	1.43	5.27	45		443	46		64	92
INITIATION Mepolizumab										
6-month evaluation Mepolizumab	21	1.71	5.53	96		81.83	16.8	2	82	89
18-month evaluation Mepolizumab	17	1.86	5.27	116		20	4	3	67	85
30-month evaluation Mepolizumab	16	2.86	4.47	157	2190	60	14	0	55	79
42-month evaluation Mepolizumab	17	2.43	4.8	119	4390	47	3.6	0	59	83
STOP mepolizumab and INITIATION dupilumab										
Dupilumab 6-month evaluation	17	2.14	5.2	32	1184	930	38	0	59	83
18-month evaluation Dupilumab	17	1.43	5.67	46	337	700	24	0	79	85
30-month evaluation Dupilumab	21	1.57	5.6	44	283	410	5	0	86	95

**Fig. 3:**
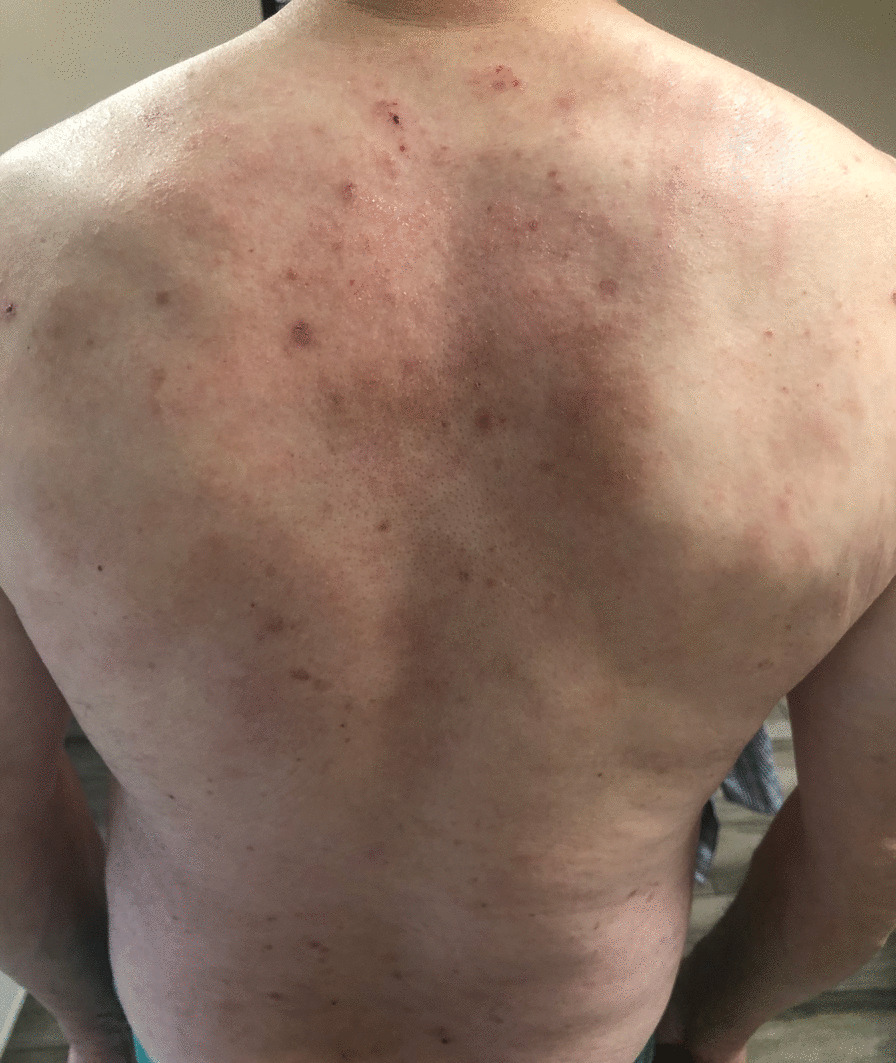
Dermatitis under treatment with mepolizumab and before starting dupilumab

Although no asthma exacerbation occurred over the last 24 months while being treated with mepolizumab and high-dose fluticasone/salmeterol, the patient developed a very aggressive and invalidating atopic dermatitis (Fig. 3), which was not satisfactorily relieved by first-step medications such as dermatocorticoids. Therefore, the dermatologist suggested to switch from mepolizumab to dupilumab (200 mg subcutaneous for 14 days) to treat the atopic dermatitis.

**Fig. 4:**
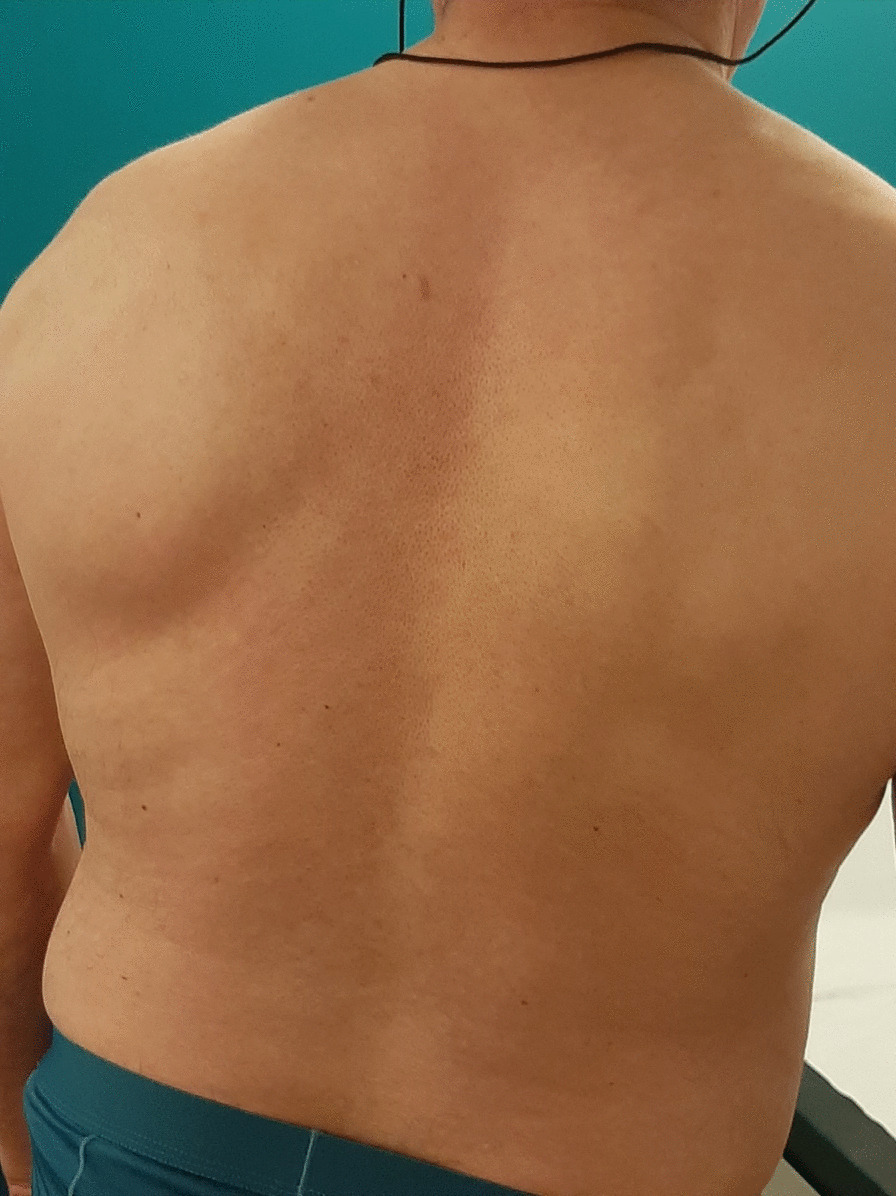
Skin lesions healing 6 months after starting dupilumab

The patient was reassessed 6 months after starting dupilumab. No exacerbation of asthma had occurred and there was a marked improvement of the skin lesions (Fig. 4). Pre- and post-bronchodilation FEV1 were measured at 59% and 83% predicted, respectively. Asthma was still not controlled, with ACT and ACQ at 17 and 2.14, respectively, and AQLQ score was 5.2 (Fig. 1). FeNO level had decreased to 32 ppb but sputum and blood eosinophils had increased to 38% and 930 µl, respectively, whereas total IgE was still high at 1187 kU/l (Fig. 2). Thus, compared with the treatment with mepolizumab, the clinical situation was stable regarding asthma control and exacerbation but markedly improved with respect to dermatitis.

Further assessments were performed after 18 months and 30 months of dupilumab treatment (Table 2). There had been no exacerbation requiring OCS over the whole period, and asthma control was obtained as reflected by ACQ < 1.5 at 18 months and ACT > 20 at 30 months. Moreover, there was a sustained improvement in FEV1 with pre- and post-bronchodilation FEV1 at 30 months reaching 86% and 95% predicted, respectively. Sputum eosinophil and blood eosinophil counts remained elevated and clearly above normal values (> 3% for sputum and > 400 µl for blood) throughout the period whereas total IgE serum and FeNO levels had markedly decreased compared with the period when the patient was receiving mepolizumab, though being still slightly elevated compared with normal values.

## Discussion

The present case describes the evolution of a severe eosinophilic atopic asthmatic who displayed clear clinical improvement in asthma control and quality of life together with substantial reduction in exacerbation after treatment with mepolizumab for 42 months (Fig. 1). The occurrence of a severe atopic dermatitis while the patient was receiving mepolizumab drove us to switch the biologic from mepolizumab to dupilumab. On dupilumab, the patient has kept good asthma control and has shown no exacerbation over a 30-month treatment period. Furthermore, with an ACQ < 1.5 at the latest visit, the patient achieved a clinical status that could be qualified as a state of remission [14]. The originality of this case resides in the concomitant detailed and sequential description of blood and airway inflammatory parameters after mepolizumab and dupilumab. It shows that dupilumab provides asthma remission without controlling systemic and airway eosinophilic inflammation.

Our case clearly shows that mepolizumab and dupilumab have a very distinct and contrasted effect on inflammatory biomarkers, supporting their different molecular mechanisms of action [7, 15, 16]. Our case illustrates the efficacy of mepolizumab in reducing exacerbation and improving asthma control through its ability to curb eosinophilic inflammation, even if the magnitude of eosinophil decrease is much greater in blood than in sputum. Both RCT trials and real-world data have shown that the magnitude of blood eosinophilic inflammation predicts the clinical response in terms of reduction of exacerbation and improvement in FEV1 [17, 18], and a recent clinical observation from our asthma clinic has indicated that sputum eosinophils may predict the possibility of achieving remission with mepolizumab treatment [19]. However, particularly intriguing is the fact that, in the months following the switch to dupilumab, asthma exacerbation was maintained to none whereas there was a massive rebound increase in both blood and sputum eosinophils. This suggests that asthma exacerbation may still be suppressed despite the persistence of intense eosinophilic inflammation. The rise in blood eosinophil after starting dupilumab had already been described in a RCT [20] but the rise in sputum eosinophils is a novel and somewhat unexpected observation since the rise in blood eosinophils was supposed to be due to a reduction of eosinophil passage through endothelium into the peripheral tissue as a consequence of reduced VCAM-1 (Vascular cell adhesion molecular 1) expression mediated by IL-4 [21, 22]. Our case clearly shows that eosinophil may still access the airways while the patient was treated with dupilumab. In keeping with our finding, a previous unpublished bronchial biopsy study showed that treatment with dupilumab did not reduce tissular eosinophils [23]. Whether eosinophil phenotype and activation state after dupilumab were similar to those before initiation of mepolizumab remains to be determined. There are arguments for heterogeneity among eosinophils, some of them playing a regulatory role instead of being proinflammatory [24]. The mechanism by which dupilumab improves asthma control and reduces exacerbation while eosinophils are persistent may be linked to an impact of IL-4 and IL-13 on mucus secretion and airway smooth muscle [22]. It is worth noting that, although persistent, the intensity of both systemic and airway eosinophilic inflammation seems to decrease over time, with sputum and blood values only slightly raised above normal after 30 months of dupilumab treatment.

Flare-up of dermatitis was the reason for the switch of biologic in our case. Why dermatitis flourished while the patient was under mepolizumab remains unclear. Whether it was related to the blocking of interleukin-5 or to the sharp reduction of OCS consumption contemporaneous of the mepolizumab treatment is unclear. It is noticeable that the dermatitis flare-up was associated with a rise in total serum IgE that contrasted with a sharp reduction in blood eosinophils. It would suggest that the IL-4 path might have been favored after blocking the effect of IL-5. In contrast, switching to dupilumab reduced total IgE and FeNO, thereby confirming what had been demonstrated in RCTs [25, 26], but initiation of dupilumab also resulted in a sharp increase in blood and, more surprisingly, sputum eosinophils. Our case indicates that treatment with anti-IL-5 and anti-IL-4/IL-13 suppresses the usual relationship between FeNO and sputum eosinophils found in large cohorts of asthmatics, including both steroid-naïve and ICS-treated patients [27, 28].

Our case not only illustrates the effectiveness of both mepolizumab and dupilumab in severe T2 high asthma, but also highlights the different mechanisms of action between the two biologics. In addition, it questions the role of eosinophils in mediating the severity of disease expression.

## Conclusion

The originality of this case resides in the description of clinical status and biomarker evolution after a sequential use of mepolizumab and dupilumab in a severe atopic eosinophilic asthmatic. It shows that mepolizumab reduces exacerbation and improves asthma control by curbing eosinophilic inflammation, whereas dupilumab provides asthma remission without controlling airway eosinophilic inflammation.

## Data Availability

All data generated or analysed during this study/case report are included in this published article (and its Additional files).
